# Myosin VI contributes to synaptic transmission and development at the *Drosophila *neuromuscular junction

**DOI:** 10.1186/1471-2202-12-65

**Published:** 2011-07-11

**Authors:** Marta Kisiel, Debolina Majumdar, Shelagh Campbell, Bryan A Stewart

**Affiliations:** 1 Department of Biology, University of Toronto Mississauga, 3359 Mississauga Rd, Mississauga, ON, L5L 1C6, Canada

## Abstract

**Background:**

Myosin VI, encoded by *jaguar *(*jar) *in *Drosophila melanogaster*, is a unique member of the myosin superfamily of actin-based motor proteins. Myosin VI is the only myosin known to move towards the minus or pointed ends of actin filaments. Although Myosin VI has been implicated in numerous cellular processes as both an anchor and a transporter, little is known about the role of Myosin VI in the nervous system. We previously recovered *jar *in a screen for genes that modify neuromuscular junction (NMJ) development and here we report on the genetic analysis of Myosin VI in synaptic development and function using loss of function *jar *alleles.

**Results:**

Our experiments on *Drosophila *third instar larvae revealed decreased locomotor activity, a decrease in NMJ length, a reduction in synaptic bouton number, and altered synaptic vesicle localization in *jar *mutants. Furthermore, our studies of synaptic transmission revealed alterations in both basal synaptic transmission and short-term plasticity at the *jar *mutant neuromuscular synapse.

**Conclusions:**

Altogether these findings indicate that Myosin VI is important for proper synaptic function and morphology. Myosin VI may be functioning as an anchor to tether vesicles to the bouton periphery and, thereby, participating in the regulation of synaptic vesicle mobilization during synaptic transmission.

## Background

While many of the molecular mechanisms that regulate the synaptic vesicle cycle are becoming understood, the methods by which vesicles move within the nerve terminal are less well known. Even though vesicle movement is an underlying assumption of most models of synaptic vesicle trafficking, it has only been demonstrated recently that the pool of vesicles is dynamic [1-3]. Understanding the mechanisms by which vesicles move within nerve terminals will inform our knowledge of both the exo- and endocytic branches of the vesicle cycle.

Two modes of transport can be envisioned: diffusion and active transport. Some studies, such as an analysis of vesicles in the recycling pool, are consistent with the notion that simple diffusion is a contributing force [4]. However, several studies indicate that a myosin-based active transport mechanism may be important [2,5,6], although there are some results to the contrary [7]. Members of the myosin-family of motor proteins share the feature of converting energy derived from ATP into molecular motion and they are used in a host of cellular processes including cell motility, cytokinesis, intracellular transport and muscle contraction.

Within the myosin superfamily of proteins, Myosin VI shares the well-conserved basic structure of other myosin proteins, however, it is the only myosin known to move towards the pointed (minus) ends of actin filaments. Myosin VI contains a unique insert present between the converter and the IQ motif of the motor domain that repositions its lever arm resulting in reverse directionality [8]. Additionally, another unique insert near the nucleotide-binding pocket of the motor domain regulates Myosin VI kinetics by restricting the accessibility of ATP to the nucleotide-binding pocket, which slows ADP release and hence, slows the dissociation of Myosin VI from actin [8]. The slow kinetic properties of Myosin VI suggest that it may function as an anchor that can link components to the actin cytoskeleton, in addition to functioning as a cargo transporter [9-12]. Myosin VI has been specifically implicated in a number of diverse cellular roles including stereocilia maintenance [13], spermatid individualization [14-16], border cell migration [17,18], nuclear transcription [19], epithelial cell-cell contacts [20,21], and regulating Golgi morphology and exocytosis [22,23]. In addition, Myosin VI is thought to have a function in the vesicle cycle in clathrin-mediated endocytosis since some splice variants of Myosin VI in human and mammalian tissues localize to clathrin-coated pits/vesicles [24]. In mammalian cells, Myosin VI has been shown to associate with endocytic vesicles following clathrin uncoating and to subsequently transport these uncoated vesicles through the actin-rich periphery to the early endosome [25]. Cells of Myosin VI knock-out mice exhibit defects in clathrin-coated vesicle formation and internalization [26]. The mechanism of Myosin VI transport in endocytosis has yet to be fully resolved as both monomers and dimers of Myosin VI have been observed to move processively upon cargo binding [9,11,27].

Although Myosin VI has received intense attention as a result of its unique directionality and its ability to perform distinct functions as a cargo transporter and anchor in the cell, relatively little is known regarding the role of Myosin VI in the nervous system [28]. Myosin VI mutant mice exhibit a decrease in the number of synapses, a decrease in dendritic spine length and an increase in the number of astrocytes as well as impaired synaptic transmission [28,29]. In *Drosophila*, Myosin VI has been shown to contribute to the asymmetric division of neural progenitor cells during embryonic nervous system development [30].

Additionally, a genetic study from our lab identified a potential role for Myosin VI as a modifier of NMJ development [31]. We were therefore interested in understanding the role of Myosin VI at this model synapse and asked how loss of *jar *affects synaptic function and development. We first observed a significant locomotor defect in severe *jar *loss of function mutants, and then we used microscopy and electrophysiology to further characterize the role of Myosin VI at the NMJ. We show novel roles for Myosin VI in maintaining proper synaptic vesicle localization to the bouton periphery and in regulating synaptic transmission.

## Results

### Myosin VI protein expression levels in *jar *loss of function mutants

**Figure 1:**
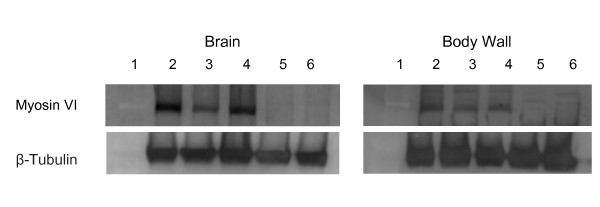
**Quantification of loss of function *jaguar *alleles**. Western blotting of third instar larval brains and body walls revealed a reduction in Myosin VI levels in *jar *loss of function mutants. Lanes for both blots are numbered as follows: 1-ladder, 2-OreR, 3- *jar*^*322*^*/+*, 4- *Df(3R)crb87-5/+*, 5- *jar*^*322*^*/Df(3R)crb87-5 *and 6- *jar*^*322*^*/Df(3R)crb87-5 MN*. The loading control used was β-Tubulin. Relative Myosin VI proteins levels were first normalized to the level of β-tubulin and then quantified. Myosin VI levels were reduced in *jar*^*322*^*/+ *and *Df(3R)crb87-5/+ *larvae compared to the control in blots of both brain and body wall samples. No bands were observed for either brain or body wall samples for *jar*^*322*^*/Df(3R)crb87-5 *and *jar*^*322*^*/Df(3R)crb87-5 MN *larvae.

The *jar *alleles used here have been previously characterized [11,32]; we quantified the Myosin VI protein levels by western blot of third instar larval brains and body walls, excluding the mouth parts. The resulting blot revealed reduced Myosin VI levels in all *jar *loss of function mutants tested (Figure 1), with virtually no Myosin VI found in the most severe *jar*^*322*^*/Df(3R)crb87-5 *zygotic and maternal null allelic combination. When the band density was measured for Myosin VI staining and normalized to the intensity of the loading control, β-tubulin, a reduction in Myosin VI levels was also observed in two different heterozygote combinations, *jar*^*322*^*/+ *and *Df(3R)crb87-5/+*, as compared to the control in blots of both brain and body wall samples, although this is less obvious on the blot. These data are representative of three separate trials of this experiment and serve to confirm the Myosin VI alleles used for this study were loss of function, consistent with previous descriptions of these alleles [32].

### Myosin VI mutants exhibit general locomotor defects

**Figure 2:**
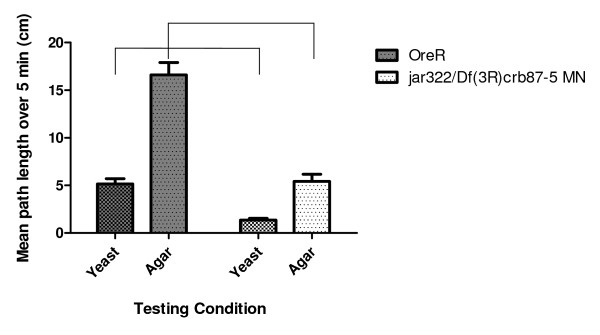
***jaguar *mutants exhibit general larval locomotor defects**. Third instar *jar*^*322*^*/Df(3R)crb87-5 MN *larvae exhibited a significant decrease in path length on both a nutritive and non-nutritive substrate compared to the control (ANOVA, *** = p < 0.0001).

Myosin VI mutant larvae were observed to be sluggish compared to their wild-type counterparts. To characterize any locomotor defects present in *jar *mutant larvae, larval path length testing was performed on the most severe *jar *mutant, *jar*^*322*^*/Df(3R)crb87-5 MN*, and a control strain. Larval path length measured over 5 minutes was significantly shorter on both a nutritive and non-nutritive substance for *jar*^*322*^*/Df(3R)crb87-5 MN *larvae (1.36 ± 0.19 cm, n = 40; 5.43 ± 0.75 cm, n = 20) compared to the control (5.16 ± 0.55 cm, n = 40; 16.61 ± 1.30, n = 20) (Two-Way ANOVA, p < 0.0001, Figure 2). Since the path lengths were shorter on both nutritive and non-nutritive substrates, these results indicate that the *jar *mutant exhibits general locomotor dysfunction as opposed to a foraging defect, wherein path length would be expected to differ from the control only on a nutritive substrate [33].

### Myosin VI contributes to proper synaptic morphology

**Figure 3:**
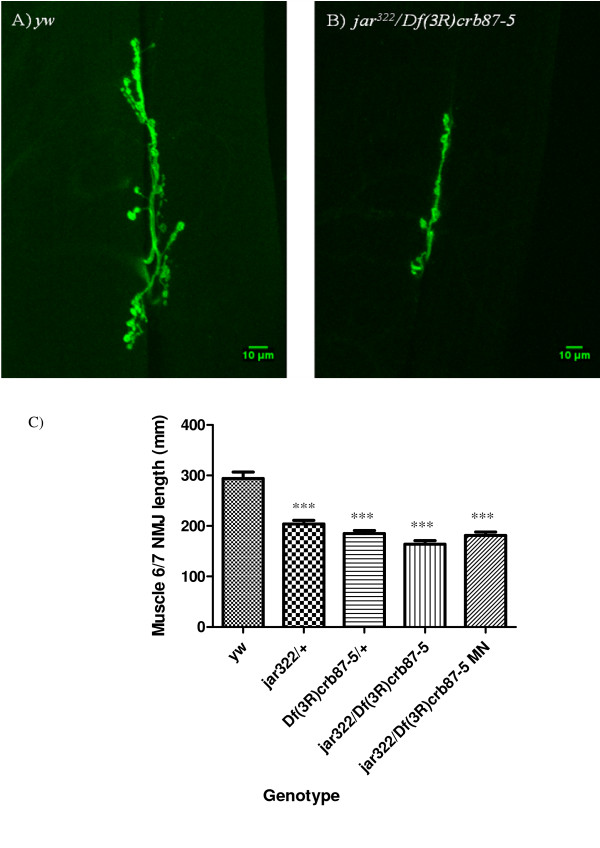
***jaguar *loss of function mutants display reduced muscle 6/7 NMJ length**. Representative NMJs on ventral longitudinal muscles 6 and 7 from third instar *Drosophila *larvae of the control (A) and the *jar *loss of function mutant, *jar*^*322*^*/Df(3R)crb87-5 *(B). All images were acquired at the same magnification using a LSM510 confocal laser microscope. Muscle 6/7 NMJ lengths for the *jar *loss of function genotypes studied were significantly shorter than the control (Dunn's Multiple Comparison Test, *** = p < 0.001) (C). Bars represent mean ± SEM.

**Figure 4:**
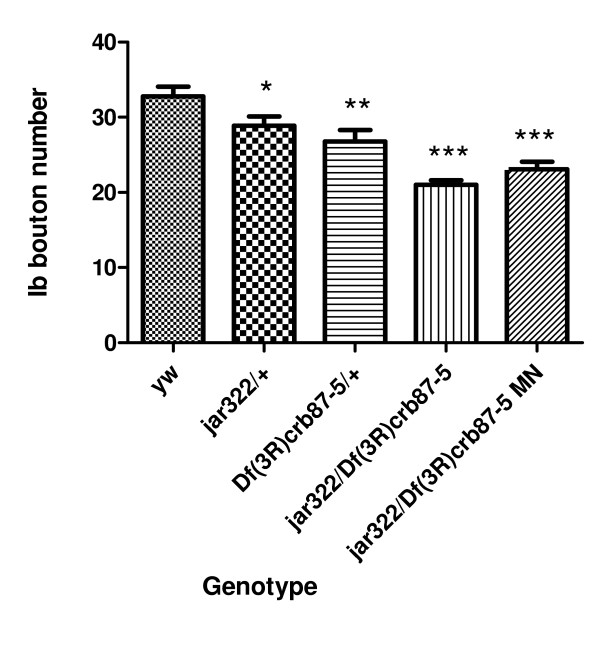
***jaguar *loss of function mutants exhibited a reduction in bouton number**. A significant decrease in Ib bouton number was observed for all *jar *loss of function mutants studied at muscle 6/7 NMJs in segments A3, A4 and A5 compared to the control (Dunnett's Multiple Comparison Test, * = p < 0.05, ** = p < 0.01, *** = p < 0.001) (D). Bars represent mean ± SEM.

Although Myosin VI is known to be important for *Drosophila *embryonic nervous system development [11,30,34], a function for Myosin VI at the NMJ has yet to be described and the locomotor defects observed in the *jar *maternal nulls mutant may reflect problems at this synapse. NMJ morphology was visualized in *jar *loss of function mutants by staining neuronal tissues with an FITC-conjugated anti-HRP antibody. Third instar larval NMJs on ventral longitudinal muscles 6 and 7 exhibited a significant decrease in NMJ length for all of the *jar *loss of function mutants studied compared to the control (Dunn's Multiple Comparison Test, p < 0.05, Figure 3). The shortest mean NMJ length was observed for *jar*^*322*^*/Df(3R)crb87-5 *larvae, 171.56 ± 2.29822 μm (mean ± SE, n *= *24 NMJs), compared to the control, 293.87 ± 12.9877 μm (n = 66 NMJs). In addition, a significant decrease in Ib bouton number was observed for *jar *loss of function mutants at muscle 6/7 NMJs (Dunnett's Multiple Comparison Test, p < 0.05) (Figure 4). On control NMJs from muscle 6/7, 32.78 ± 1.3025 boutons (mean ± SE, *n = *58 NMJs) were observed; whereas these synapses in *jar*^*322*^*/Df(3R)crb87-5 *larvae had a mean number of 21.02 ± 0.60573 boutons (*n = *58 NMJs).

**Figure 5:**
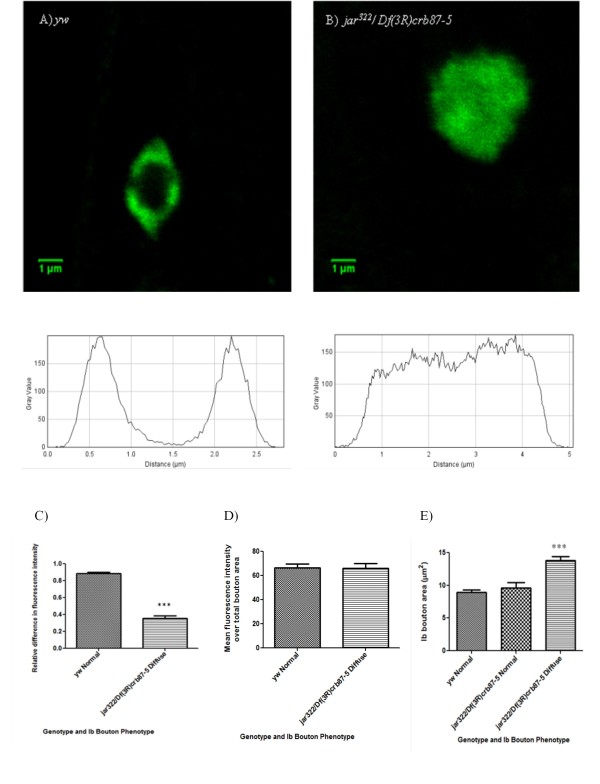
**Synaptic vesicles are mislocalized at *jaguar *mutant boutons as revealed by immunostaining against synaptotagmin**. A representative control Ib bouton and mutant *jar*^*322*^*/Df(3R)crb87-5 *Ib bouton are shown with their corresponding plot profiles (A and B). There was a significant reduction in relative difference in fluorescence intensity across *jar*^*322*^*/Df(3R)crb87-5 *mutant boutons (n = 13) compared to the control boutons (n = 25; Unpaired T-test, *** = p < 0.001) (C). Mean fluorescence intensity did not differ significantly between the mutant (n = 48) and the control (n = 64; Unpaired T-test, p > 0.05) (D). Diffuse *jar*^*322*^*/Df(3R)crb87-5 *boutons (n = 53) were significantly larger than control boutons (n = 64) and *jar*^*322*^*/Df(3R)crb87-5 *boutons with a normal, doughnut-shaped distribution of synaptotagmin (n = 27, Dunn's Multiple Comparison Test, *** = p < 0.001) (E). Boutons used for this study were from muscle 6/7 NMJs in segments A3, A4 and A5. Bars represent mean ± SEM.

To further investigate the role of Myosin VI in regulating synaptic morphology, staining of the integral vesicle membrane protein Synaptotagmin I was used to visualize synaptic vesicle localization in the boutons of control and *jar *loss of function mutant larvae. Ultrastructural studies have shown that vesicles are localized to the periphery of boutons, with the centre either void or partially occupied by mitochondria [35]; this gives rise to a characteristic torus (doughnut)-shaped synaptotagmin pattern, with vesicles concentrated in an outer ring of the bouton when viewed with the light microscope [36]. Surprisingly, visualization of synaptotagmin staining in *jar *mutant boutons revealed a disruption in proper vesicle localization. The majority of *jar*^*322*^*/Df(3R)crb87-5 *Ib boutons exhibited diffuse synaptotagmin localization across the entire bouton area, rather than the characteristic torus-pattern. The differences in synaptotagmin distribution are illustrated by representative control and mutant *jar*^*322*^*/Df(3R)crb87-5 *boutons, shown with their corresponding plot profiles (Figure 5A and 5B).

To quantify these observed differences in synaptotagmin localization we calculated the relative difference in fluorescence intensity across a bouton's plot profile [(max intensity-min intensity)/max intensity]. Relative difference in fluorescence intensity was significantly lower across *jar*^*322*^*/Df(3R)crb87-5 *mutant boutons compared to control boutons (Unpaired T-test, p < 0.001) (Figure 5C). However, there was no significant difference in mean fluorescence intensity over total bouton area for both genotypes (Unpaired T-test, p > 0.05) (Figure 5D). Interestingly, diffuse *jar*^*322*^*/Df(3R)crb87-5 *boutons were found to be significantly larger than control boutons and *jar*^*322*^*/Df(3R)crb87-5 *boutons with a normal, torus-shaped distribution of synaptotagmin (Dunn's Multiple Comparison Test, p < 0.001) (Figure 5E).

**Figure 6:**
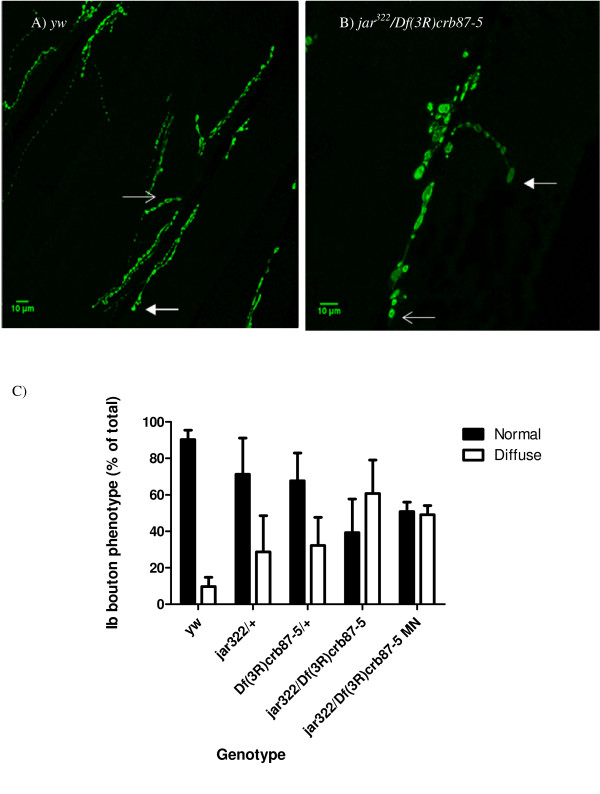
**Boutons exhibiting vesicle mislocalization increased corresponding to the severity of *jaguar *loss of function**. Immunostaining against synaptotagmin of NMJs on muscles 6/7 of control and *jar*^*322*^*/Df(3R)crb87-5 *third instar larvae revealed different proportions of boutons exhibiting normal and diffuse synaptotagmin staining (A and B). Images were taken at different magnifications to visualize the entire NMJ. Open arrows indicate the normal, doughnut-shaped pattern of synaptotagmin localization and closed arrows indicate the diffuse pattern of synaptotagmin localization. Scoring the synaptotagmin distribution phenotype revealed a reduction in the percentage of normal boutons and an increase in the percentage of diffuse boutons in *jar *loss of function mutants compared to the control (C). Number of boutons analyzed were *yw *n = 48, *jar322/+ *n = 38, *Df(3R)crb87-5/+ *n = 28, *jar*^*322*^*/Df(3R)crb87-5 *n = 58 and *jar*^*322*^*/Df(3R)crb87-5 MN *n = 18. Boutons used for this study were from muscle 6/7 NMJs in segments A3, A4 and A5. Bars represent mean ± SEM.

To quantify the population of boutons displaying abnormal synaptotagmin distribution, Ib boutons were scored as normal or diffuse for control and *jar *mutant muscle 6/7 NMJs in third instar larvae. This protocol revealed an increase in the percentage of diffuse Ib boutons for *jar *mutant NMJs compared to control NMJs (Figure 6). A Chi Square goodness of fit test revealed that all *jar *mutants significantly differed from the control in number of normal and diffuse Ib boutons (p < 0.05). Taken together these data indicate that Myosin VI contributes to proper synaptic development. Specifically, the unexpected diffuse synaptotagmin staining over the Ib bouton centre observed in *jar *mutants suggests that Myosin VI plays a role in maintaining normal peripheral vesicle localization.

### Myosin VI plays a role in basal synaptic transmission

As the localization of synaptic vesicles is known to be important in basal synaptic function [37], the vesicle mislocalization phenotype observed at *jar *mutant synapses may be associated with impaired synaptic transmission. To characterize synaptic physiology at *jar *mutant synapses, synaptic transmission was assessed by measuring nerve-evoked and spontaneous transmitter release at larval NMJs. Low frequency stimulation allows us to assess the state of the readily releasable pool of vesicles, whereas higher frequency stimulation allows us to probe the recruitment of vesicles from the reserve pool. The ability of the neuron to maintain transmission under sustained high frequency stimulation also allows us to measure the endocytotic ability of the synapse.

**Figure 7:**
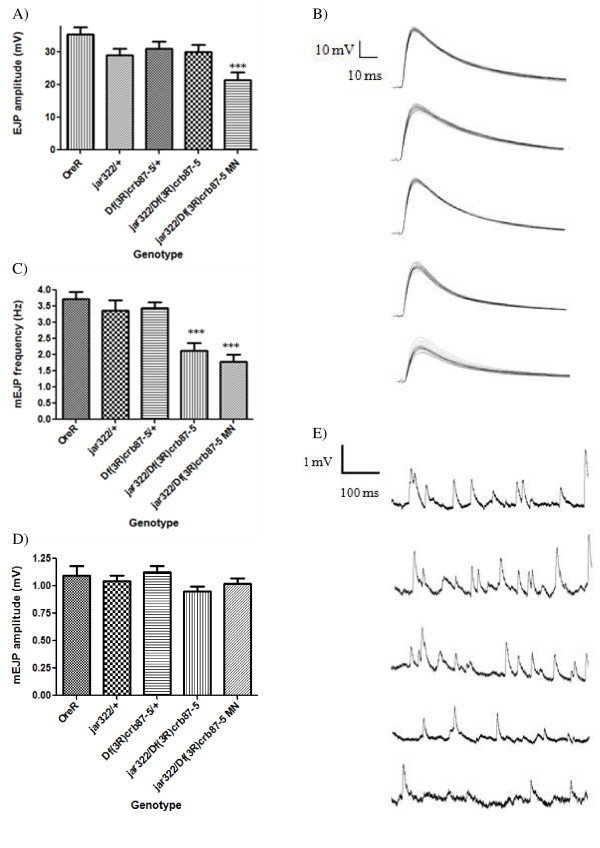
***jaguar *loss of function mutants exhibited defects in basal synaptic transmission**. Electrophysiological recordings from muscles 6/7 of third instar larvae revealed EJP amplitude was significantly reduced in the most severe *jar *mutant, *jar*^*322*^*/Df(3R)crb87-5 MN*, compared to the control (Dunnett's Multiple Comparison Test, *** = p < 0.001) (A). Sample traces of EJP recordings taken at 1 Hz, shown in the same order as listed for the graphs (B). mEJP frequency was significantly lower for *jar*^*322*^*/Df(3R)crb87-5 *and *jar*^*322*^*/Df(3R)crb87-5 MN *larvae compared to the control larvae (Dunnett's Multiple Comparison Test, *** = p < 0.001) (C). There was no significant difference in mEJP amplitude between *jar *mutants and the control (ANOVA, p > 0.05) (D). Sample traces of electrophysiological recordings from muscles 6/7 over 2 seconds, shown in the same order as listed for the graphs (E). Bars represent mean ± SEM.

Low frequency nerve-evoked responses were collected at 1 Hz stimulation and the mean EJP amplitude was calculated from 16 stimuli per NMJ. *jar *mutants lacking a maternal contribution of Myosin VI, *jar*^*322*^*/Df(3R)crb87-5 MN*, exhibited a significantly lower mean EJP amplitude (21.34 ± 2.4572 mV, n = 16) than the control (35.47 ± 2.2311 mV, n = 12; Dunnett's Multiple Comparison Test, p < 0.001) (Figure 7A).

Recordings of spontaneous vesicle release, collected for 1 to 2 minutes, were used to calculate mEJP amplitude and frequency. A reduction in mEJP frequency was observed in the more severe *jar *loss of function mutants, *jar*^*322*^*/Df(3R)crb87-5 *and *jar*^*322*^*/Df(3R)crb87-5 MN *(2.12 ± 0.238 Hz, n = 17 and 1.79 ± 0.2104 Hz, n = 16 respectively), compared to the control (3.73 ± 0.2092 Hz, n = 14; Dunnett's Multiple Comparison Test, p < 0.001) (Figure 7C). Average mEJP amplitude was found to be approximately 1 mV for all genotypes studied, with no significant differences between them (ANOVA, p > 0.05) (Figure 7D).

### Myosin VI affects short-term synaptic plasticity

**Figure 8:**
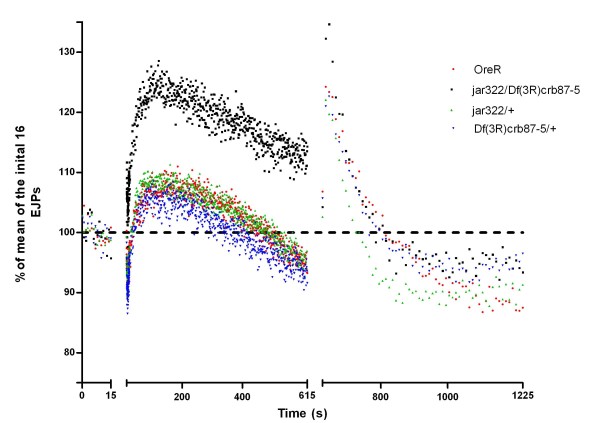
**High frequency stimulation in 1 mM Ca**^**2+ **^**saline revealed enhanced potentiation in *jar***^***322***^***/Df(3R)crb87-5 *mutants**. Data is shown as a percent of the average of the first 16 evoked junctional potentials (EJPs) recorded at 1 Hz. This baseline recording of 16 EJPs at 1 Hz was followed by a stimulation of 10 Hz recorded over 10 minutes and terminated with a 10 minute recording at 0.1 Hz. Following the first 25 seconds from the onset of 10 Hz stimulation, every tenth recording is shown. A significant enhancement in EJP amplitude was observed for *jar*^*322*^*/Df(3R)crb87-5 *larvae during the first three minutes of the high frequency protocol compared to the control (Dunnett's Multiple Comparison Test, p < 0.001).

To further characterize synaptic function at *jar *mutant synapses, a high frequency stimulation protocol was used to examine changes in synaptic plasticity. The high frequency stimulation protocol was 16 EJPs at 1 Hz, followed by 10 Hz stimulation for 10 minutes, concluding with 0.1 Hz stimulation for 10 minutes. In 1 mM extracellular Ca^2+ ^HL3-saline, the typical pattern of synaptic response to 10 Hz stimulation began with rapid depression of EJP amplitude, likely due to the depletion of the readily releasable pool (RRP) [38,39]. This was followed by an increase in EJP amplitude, corresponding to mobilization of the reserve pool (RP) due to the presence of residual Ca^2+ ^in the neuronal cytoplasm [6,38], and a subsequent steady decline with continuing high frequency stimulation. For analysis, EJP amplitudes were normalized to the average of the first 16 EJPs collected at 1 Hz for each recording. No significant differences in depression at the onset of high frequency stimulation were observed between the genotypes (ANOVA, p > 0.05) (Figure 8). The enhancement in EJP amplitude following the initial depression was significantly greater for *jar*^*322*^*/Df(3R)crb87-5 *larvae than the control larvae (124.14 ± 5.97126%, n = 11 and 107.49 ± 2.1921%, n = 10 respectively; Dunnett's Multiple Comparison Test, p < 0.001) (Figure 8). There were no significant differences in maximum EJP amplitude for the *jar *loss of function heterozygotes relative to the control.

Following high frequency stimulation, post-tetanic potentiation (PTP) is observed as an enhancement of EJP for a short period of time. PTP is presynaptic in origin, caused by an increase in neurotransmitter quanta release, which is attributed to calcium release from intracellular stores that was accumulated during high frequency stimulation [39,40]. No significant difference was found in the extent of PTP at 0.1 Hz for the genotypes studied (ANOVA, p > 0.5) (Figure 8). In addition, there was no significant difference in the amplitude of the last EJP measured at 0.1 Hz stimulation between genotypes (ANOVA, p > 0.5) (Figure 8).

**Figure 9:**
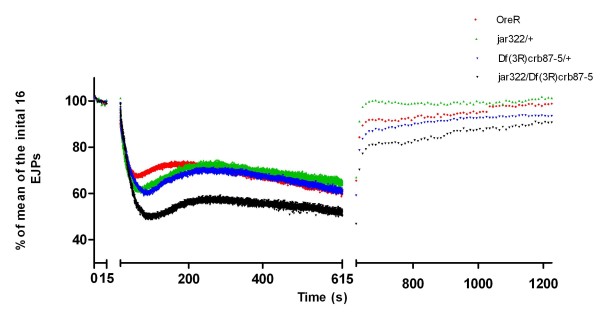
**High frequency stimulation in 10 mM Ca**^**2+ **^**saline revealed enhanced depression in *jar***^***322***^***/Df(3R)crb87-5 *mutants**. Data is shown as a percent of the average of the first 16 evoked junctional potentials (EJPs) recorded at 1 Hz. This baseline recording of 16 EJPs at 1 Hz was followed by a stimulation of 10 Hz recorded over 10 minutes and terminated with a 10 minute recording at 0.1 Hz. Following the first 25 seconds from the onset of 10 Hz stimulation, every tenth recording is shown. The initial depression in EJP amplitude measured relative to EJP amplitude at the onset of high frequency stimulation was significantly greater for *jar*^*322*^*/Df(3R)crb87-5 *larvae than the control larvae (ANOVA, p < 0.01).

To further challenge the synapse, the same high frequency stimulation protocol was carried out in 10 mM (supraphysiological) Ca^2+ ^saline. This produced a rapid depression in EJP amplitude at the onset of 10 Hz simulation, likely due to vesicle depletion in response to high calcium concentrations [39]. The initial depression was followed by a slight recovery and then a continuing decline in EJP amplitude for the remainder of the 10 Hz stimulation. The initial depression in EJP amplitude measured relative to EJP amplitude at the onset of high frequency stimulation was significantly greater for *jar*^*322*^*/Df(3R)crb87-5 *larvae than the control larvae (0.63 ± 0.04, n = 12 and 0.45 ± 0.02, n = 8 respectively; ANOVA, p < 0.01) (Figure 9). There were no significant differences in initial depression among the other genotypes tested (ANOVA, p > 0.05). Recovery of EJP amplitude at 10 Hz stimulation was quantified as a percent increase from the lowest EJP amplitude during the initial depression to the largest EJP amplitude observed during the recovery period. There was no difference in recovery among control and *jar *loss of function larvae following 10 Hz stimulation (ANOVA, p > 0.05). Additionally, no difference in recovery of EJP amplitude was found among genotypes at 0.1 Hz stimulation (ANOVA, p > 0.05)

## Discussion

This work has demonstrated a novel role for Myosin VI in proper synaptic development, vesicle localization, and synaptic transmission at the *Drosophila *NMJ. Severe *jar *loss of function mutants exhibited significant defects in larval locomotor behavior prompting our investigations of Myosin VI at the NMJ. Imaging of synaptic morphology revealed that Myosin VI is important for NMJ development: a reduction in NMJ length and a decrease in bouton number were observed at *jar *loss of function mutant nerve terminals.

Although Myosin VI function in the vesicle cycle has been implicated in mammalian cells, this report provides the first evidence that Myosin VI is important for maintaining normal peripheral vesicle localization at the bouton [25]. In *Drosophila*, there are four types of boutons, which are the sites of neurotransmitter release at the NMJ, and they differ in their morphological and chemical properties [41]. Of interest for this study were the largest synaptic boutons found at type I axon terminals, which are present at all NMJs of mature larvae [42]. Visualization of synaptotagmin staining using confocal imaging revealed a mislocalization of synaptic vesicles in *jar *mutant boutons. An increasing number of *jar *mutant boutons, corresponding to the severity of Myosin VI loss of function, were found to exhibit diffuse staining over the entire bouton area as opposed to the doughnut-shaped staining pattern present in control boutons. Bouton centre occupancy has previously been observed at *Drosophila *NMJs of larvae lacking synapsin, a phosphoprotein that reversibly associates with vesicles, using FM1-43 loading under low frequencies [43]. EM analysis confirmed that in synapsin knockouts there was a spread of vesicles into the bouton centre, accompanied by a reduction in the size of the reserve pool [44]. Thus, synapsin is thought to function in maintaining the peripheral distribution of vesicles in Ib boutons [44]. Likewise, the unexpected diffuse synaptotagmin staining of *jar *mutant boutons suggests Myosin VI participates in restricting vesicles to the bouton periphery. It is possible that Myosin VI is functioning as a regulator of the actin cytoskeleton at the synapse. Mutant studies have revealed that the presynaptic actin cytoskeleton is required for proper synaptic morphogenesis [45]. Myosin VI has already been shown to function in regulating the actin cytoskeleton during the process of spermatid individualization, by acting either as a structural cross-linker or as an anchor at the front edge of the actin cone, and during nuclear divisions in the syncytial blastoderm [15,46]. However, live imaging of actin dynamics at the synaptic boutons revealed no major defects in the actin cytoskeleton at *jar *loss of function mutant nerve terminals.

To assess whether the morphological defects in vesicle localization observed at *jar *mutant synapses impact synaptic transmission, electrophysiological assays with different stimulation paradigms were used to recruit vesicles from different functional pools. Our data add to the knowledge of this protein's physiological role at synapses. Myosin VI mutant mouse hippocampal neurons exhibit defects in the internalization of the α-amino-3-hydroxy-5-methyl-4-isoxazole propionic acid-type glutamate receptor, responsible for fast glutamatergic transmission, suggesting Myosin VI normally plays a role in AMPAR endocytosis [28]. In addition, basal synaptic transmission is reduced in Myosin VI deficient mouse hippocampal slices compared to wild-type controls [29]. Electrophysiological experiments also indicate that Myosin VI mediates glutamate release induced by brain-derived neurotrophic factor, which is known to modulate synaptic transmission and plasticity in the mammalian central and peripheral nervous system [29].

Our study is the first to show that Myosin VI's role in synaptic transmission involves mobilization of vesicles from different functional pools, indicating that Myosin VI is important for synaptic plasticity. At the *Drosophila *NMJ, three pools of vesicles with differential release properties have been identified using FM1-43 staining loaded by various stimulation protocols ([47], reviewed by [48]). The immediately releasable pool (IRP), representing approximately 1% of all vesicles at the NMJ, consists of vesicles docked and primed at active zones for immediate release and experiences rapid depletion within a few stimuli [48,49]. The readily releasable pool (RRP), making up 14 to 19% of all vesicles at the NMJ, is mobilized by moderate stimulation of ≤3 Hz and maintains exo/endocytosis at these stimulation frequencies [49]. The reserve pool (RP) represents the vast majority of vesicles, 80 to 90%, and is mobilized upon depletion of the RRP [49]. Recruitment from the RP occurs with high frequency stimulation of ≥10 Hz [50]. Spontaneous release was reduced in the most severe *jar *loss of function mutants. Evoked response at 1 Hz stimulation was also reduced in the *jar *maternal null mutant. Although less severe *jar *mutants exhibited a significant decrease in bouton number, they did not experience an accompanying reduction in evoked potential amplitude at low frequency stimulation, suggesting that other homeostatic mechanisms are important for maintaining synaptic strength [51]. The impaired synaptic response in the *jar *maternal null mutant may be due to a reduction in the probability of RRP vesicle release or in RRP size. If Myosin VI functions to anchor synaptic vesicles, it may act on the RRP to ensure vesicles are localized in manner that makes them readily available for release. Thus, in *jar *maternal null mutants the reduction in EJP amplitude may occur because a significant number of vesicles were displaced from areas of higher probability release. Alternately, RRP pool size may be reduced at *jar *mutant synapses.

Different synaptic vesicle pool properties, such as rate of recruitment of the RP in response to high frequency stimuli, may translate to changes in short-term synaptic plasticity [37]. The increase in EJP amplitude observed at 10 Hz stimulation in 1 mM Ca^2+ ^saline may be attributable to enhanced mobilization of the RP for *jar*^*322*^*/Df(3R)crb87-5 *NMJs [44]. Filamentous actin has been implicated in RP mobilization as cytochalasin D, an inhibitor of actin polymerization, has been shown to reduce RP dynamics [47]. This suggests translocation from the RP to the RRP may be mediated by an actin-based myosin motor protein. If Myosin VI functions as a synaptic vesicle tether to regulate recruitment from the RP pool, RP vesicles would be more readily mobilized and transitioned into the RRP upon high frequency stimulation in *jar *loss of function mutants. Consistent with the idea that RP vesicles were more rapidly incorporated into the RRP, a greater initial depression is observed at *jar*^*322*^*/Df(3R)crb87-5 *mutant synapses during high frequency stimulation in 10 mM Ca^2+ ^saline corresponding to the depletion of vesicles at high calcium concentrations. Taken together, the data suggest that Myosin VI mediates synaptic transmission and short-term plasticity by regulating the mobilization of synaptic vesicles from different functional pools. In mammalian cells, Myosin VI has been implicated as a mediator of vesicle endocyctosis and has been shown to transport uncoated vesicles through the actin-rich periphery to the early endosome [25]. Our experiments, however, indicate that endocytosis is not likely affected at *jar *mutant synapses. Typically, endocytotic mutants are unable to maintain synaptic transmission in response to high frequency stimulation [52,53], whereas our Myosin VI loss of function mutants exhibited enhanced EJP amplitude observed at 10 Hz stimulation in 1 mM Ca^2+ ^saline. Additional experiments are required to confirm that Myosin VI is functioning as a vesicle tether. Fluorescence recovery after photobleaching analysis can be used to examine the effect of Myosin VI on synaptic vesicle mobility. If Myosin VI is functioning as a vesicle tether, synaptic vesicle mobility is expected to be increased in *jar *mutants compared to controls.

## Conclusions

In summary, the present work shows that Myosin VI is important for proper synaptic morphology and physiology at the *Drosophila *NMJ. Myosin VI function in peripheral vesicle localization at the bouton may underlie its contribution to basal synaptic transmission and expression of synaptic plasticity. Future work will address the mechanism by which Myosin VI performs its roles at the synapse, whether as a vesicle tether or by some other involvement in vesicle trafficking.

## Methods

### Drosophila stocks

All fly strains and crosses were maintained on Bloomington standard medium (http://flystocks.bio.indiana.edu/Fly_Work/media-recipes/bloomfood.htm) supplemented with yeast paste at room temperature. The Myosin VI loss of function alleles used in this study were *jar*^*322 *^and *Df(3R)crb87-5 *(maintained as stocks over *Tm3, Sb Ser GFP*). *jar*^*322 *^is a null allele that deletes the entire Myosin VI coding region and some of the neighboring gene, CG5706 [32]. When homozygous, *jar*^*322 *^is lethal in first or second instar larvae due to the loss of CG5706 function [32]. *Df(3R)crb87-5 *is a deletion that removes most of the amino acid coding sequences of the *jar *gene, through to exon 13 of 17 [32]. *jar*^*322*^*/Df(3R)crb87-5 *animals were produced by crossing together *jar*^*322*^*/Tm3, Sb Ser GFP *and *Df(3R)crb87-5/Tm3, Sb Ser GFP *flies, and then selecting against the GFP balancer chromosomes. Myosin VI maternal null animals, designated *jar*^*322*^*/Df(3R)crb87-5 MN*, were generated by crossing *jar*^*322*^*/Df(3R)crb87-5 *females to *jar*^*322*^*/Tm3, Sb Ser GFP *males, and then selecting non-fluorescent larvae. Larvae from *yw *flies were used as a control for staining experiments, and from Oregon R (*OreR*) flies for electrophysiological experiments.

### Western blots

Protein extracts were prepared in ice-cold homogenization buffer with proteinase inhibitor. Four body walls or ten brains from third instar larvae per genotype were used. The larval preparations were manually homogenized and pulsed in the centrifuge. This was followed by the addition of 0.5 M Dithiotreitol and 2x loading buffer. Larval preparations were heated at 100°C for 5 minutes prior to loading. Samples were run on a 6% SDS PAGE gel using modified procedures from [54]. Gels were run for 1.5 hours at 70 V, followed by electropheretic transfer to polyvinylidene difluoride membrane at 350 mA for 55 minutes. Membranes were incubated with 1:20 mouse anti-myosin VI antibody (gift from Kathy Miller) and 1:1000 anti-tubulin antibody (Hybridoma Bank, University of Iowa). HRP-conjugated goat anti-mouse antibody (BIORAD) was used at a dilution of 1:3000. Resultant bands were visualized using STORM Scanner Control (Molecular Dynamics). The stained bands were now visible for further analysis using Image J. Relative protein levels were quantified and compared.

### Test of general larval locomotion

Larval path length testing was used to assess changes in general larval locomotion in *jar*^*322*^*/Df(3R)crb87-5 MN *larvae and were performed as described by [55]. Briefly, individual third instar larvae (96 ± 2 h posthatching) were placed onto a layer of yeast paste in a circular well. Each well was covered with a Petri-dish lid and after 5 minutes, the paths traveled by the larvae were traced onto the Petri lids. These path lengths were used to calculate the mean path length in ImageJ for the mutant and control larvae, which were tested concurrently. Similarly, path length was tested on a non-nutritive substrate, 0.4% agar. Total distance traveled on agar was measured by placing individual third instar larvae (96 ± 2 h posthatching) in the center of agar-coated Petri-dishes, which were then covered with the Petri-dish lids. After 5 minutes, the path traveled by the larvae was traced onto the Petri lids and quantified as described above.

### Immunocytochemistry

For immunostaining, third instar larvae were dissected in HL3 saline, fixed in 4% EM grade formaldehyde in phosphate buffered saline (PBS) for 15 minutes and then transferred to an Eppendorf tube with 5 to 7 other larvae. For NMJ visualization, larvae were stained with FITC-conjugated goat anti-HRP antibody, (1:1000, MP Biomedical; Solon, OH), as described by [56]. For synaptic vesicle labeling, larvae were stained with 1:1000 rabbit polyclonal anti-synaptotagmin antibody, followed by the secondary antibody, AlexaFluor^® ^488 goat anti-rabbit IgG, (1:1000, Invitrogen), as outlined by [1]. Following staining, larvae were mounted onto slides in Vectashield (Vector Laboratories; Burlington, ON).

Images were obtained using a LSM510 (Carl Zeiss) confocal laser microscope with a 200 mW Argon laser. To image NMJs, Z-sections were collected for NMJ 6/7, 2 per larvae, at 1 μm intervals through the 20x air lens at 2 times magnification. These images were projected onto a single plane and NMJs were measured (scale: 2.223 pixels/μm) using Image J. For synaptotagmin staining, images for individual boutons were obtained under constant imaging parameters, including gain, zoom, pinhole size and laser output, to allow for proper comparative analysis. To analyze synaptotagmin staining in individual boutons, a rectangular cross section of the bouton was taken and a plot profile was generated for this region using Image J.

### Electrophysiology

Wandering third instar larvae were dissected in HL3 saline to which calcium was added for a final concentration of 1 mM [57]. Intracellular electrophysiological recordings for muscle 6 or 7 recordings were taken from abdominal segments A3, A4 and A5, and analyzed as described by [58]. All muscles selected for analysis had initial resting potentials between -60 and -75 mV. Baseline synaptic transmission was characterized by recording miniature excitatory junctional potentials (mEJPs) for 1 to 2 minutes and 16 evoked junctional potentials (EJPs) stimulated at 1 Hz. High frequency stimulation was performed on larvae bathed in either 1 mM Ca^2+ ^HL3 or 10 mM Ca^2+ ^modified HL3, the composition of which is as follows (in mM): 70 NaCl, 5 KCl, 10 MgCl_2_, 10 NaHCO_3_, 10 CaCl_2_, 115 sucrose, 5 Trehalose, and 5 HEPES (pH 7.2). The stimulation protocol used for high frequency recordings was 16 EJPs at 1 Hz, followed by 10 Hz stimulation for 10 minutes, and concluded with 0.1 Hz stimulation for 10 minutes. Muscle resistance was recorded for both protocols to ensure it was within normal range, 5 to 10 mΩ [59]. In all experiments, a maximum of two recordings were used per larvae and a minimum of 8 larvae were used.

### Statistical analysis

All statistical analyses were performed in GraphPad Prism Software 5. A significance level of p < 0.05 was used for all experiments.

## Competing interests

The authors declare that they have no competing interests.

## Authors' contributions

The experiments were conceived and designed by MK and BAS; MK performed all experiments, analyzed the data, and assembled figures; DM and SC performed initial observations of *jar *mutant phenotypes; MK and BAS wrote and edited the manuscript. All authors have read and approved the final manuscript.
